# Intracranial baroreflex is attenuated in an ovine model of renovascular hypertension

**DOI:** 10.1038/s41598-021-85278-3

**Published:** 2021-03-12

**Authors:** Sydney Vari, Sarah-Jane Guild, Bindu George, Rohit Ramchandra

**Affiliations:** 1 Cardiovascular Autonomic Research Cluster, Department of Physiology, The University of Auckland, Auckland, 1040 New Zealand; 2 Auckland Bioengineering Institute, Auckland, New Zealand

**Keywords:** Cardiology, Medical research

## Abstract

We have previously shown that elevations in intracranial pressure (ICP) within physiological ranges in normotensive animals increase arterial pressure; termed the intracranial baroreflex. Hypertension is associated with alterations in reflexes which maintain arterial pressure however, whether the intracranial baroreflex is altered is not known. Hence, in the present study, we tested the hypothesis that in hypertension, physiological increases in ICP would not be accompanied with an increase in arterial pressure. Renovascular hypertension was associated with no change in heart rate, renal blood flow or ICP levels compared to the normotensive group. ICV infusion of saline produced a ramped increase in ICP of 20 ± 1 mmHg. This was accompanied by an increase in arterial pressure (16 ± 2 mmHg) and a significant decrease in renal vascular conductance. ICV infusion of saline in the hypertensive group also increased ICP (19 ± 2 mmHg). However, the increase in arterial pressure was significantly attenuated in the hypertensive group (5 ± 2 mmHg). Ganglionic blockade abolished the increase in arterial pressure in both groups to increased ICP. Our data indicates that physiological increases in ICP lead to increases in arterial pressure in normotensive animals but this is severely attenuated in renovascular hypertension.

## Introduction

The cerebral circulation has a limited ability to expand within the cranium to accommodate any increase in blood volume. A key determinant of brain blood flow is the cerebral perfusion or supply pressure to the brain. Intracranial pressure (ICP) is the pressure within the cranium and it determines cerebral perfusion pressure (calculated as the difference between arterial pressure and ICP). An increase in ICP reduces cerebral perfusion and blood delivery to the brain. It is already known that large pathological rises in ICP (when ICP reaches over 25 mmHg) produces an increase in arterial pressure, bradycardia and respiratory irregularities termed the “Cushing response”^1^. In addition to these pathological changes in ICP, a role for normal physiological changes in ICP (between 2–20 mmHg) has also been suggested previously^2^, and this is termed the intracranial baroreflex. Previous studies by us in sheep^3^ and others in mice and humans^4,5^ have shown that increases in ICP within physiological ranges leads to an increase in arterial pressure. It is likely that this increase in ICP is sensed by astrocytes in the brainstem^4^, which leads to an increase in sympathetic nerve activity^3^, thereby mediating the pressor response. The current paradigm is that physiological changes in ICP (below 20 mmHg) alter arterial pressure via the intracranial baroreflex and then larger pathological changes in ICP (> 25 mmHg) alter arterial pressure via the Cushing’s reflex.

An inability to maintain cerebral perfusion has been suggested as one causative factor mediating the pathophysiology of a number of neurodegenerative diseases including mild cognitive impairment, Alzheimer’s disease, and vascular dementia^6^. In this context, hypertension, particularly in mid-life, is a known risk factor for neurodegenerative conditions^7,8^. Numerous cross-sectional studies have shown that hypertensive patients have lower cerebral perfusion than their age-matched normotensive controls^9–11^. Collectively these studies suggest that cerebral perfusion is low in hypertensive patients however it is not known if the putative response to challenges to cerebral perfusion via increases in ICP i.e. the intracranial baroreflex, is altered in hypertension. Hypertension is associated with alterations in other reflexes which maintain arterial pressure and perfusion of organs such as the arterial baroreflex^12,13^ and the peripheral chemoreflex^14,15^. Any putative attenuation of the intracranial baroreflex would mean that increases in ICP would not be buffered such that cerebral perfusion would decrease.

Hence, in the present study we investigated the role of the intracranial baroreflex in hypertension. We utilized a large animal model (sheep) since the pressure within the cranium as well as the ICP dynamics more closely resembles that seen in humans^16^. The two-kidney, one-clip model of hypertension was chosen since we were interested in the response of the contralateral (non-clipped) kidney to hypertension. Specifically, we tested the hypothesis that physiological increases in ICP in a group of conscious hypertensive sheep would not be accompanied with an increase in arterial pressure compared to the normotensive group.

## Results

**Figure 1:**
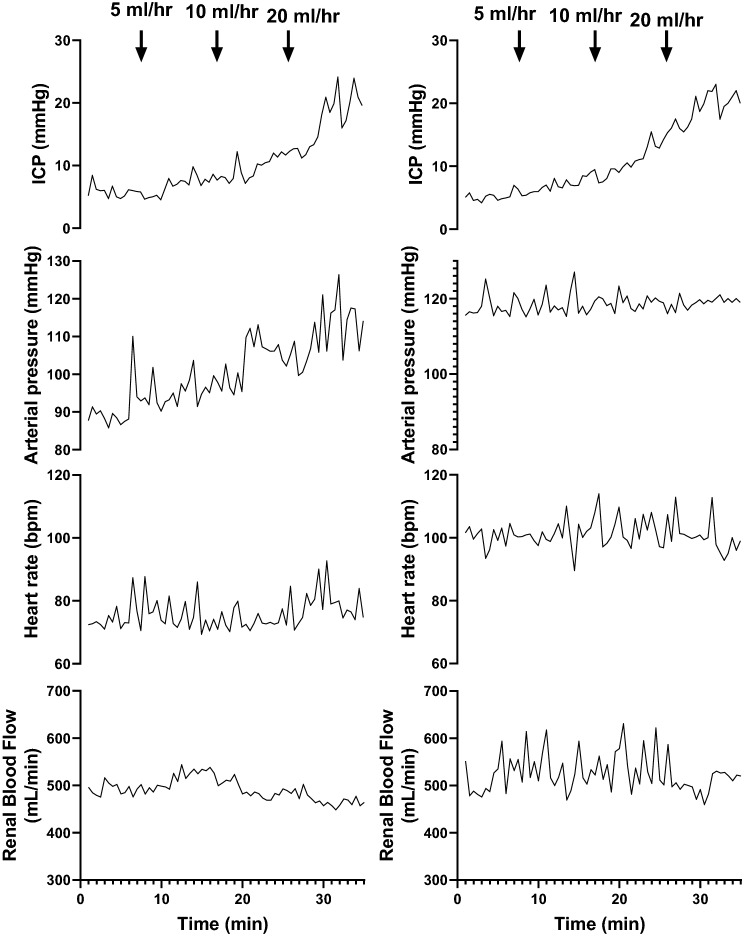
Representative example of intracranial pressure (ICP), arterial pressure, heart rate and renal blood flow showing the temporal nature of the response when ICV saline is infused in one normotensive (left) and one hypertensive (right) animal. Arrows show the start of each infusion rate.

**Figure 2:**
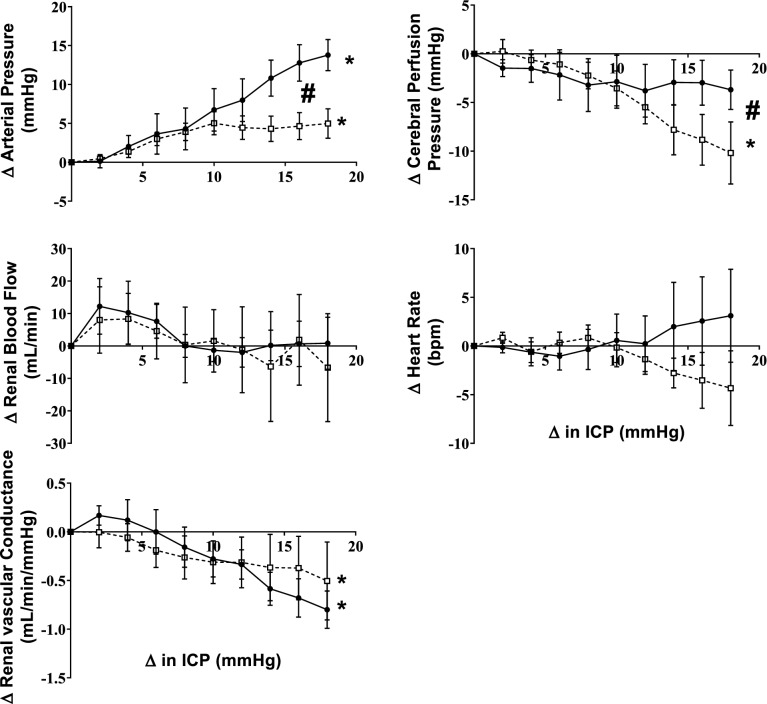
Response of arterial pressure, cerebral perfusion pressure, heart rate, renal blood flow and renal conductance to increased ICP in the normotensive (n = 6) and the hypertensive group (n = 6). All parameters are plotted as an absolute change from baseline. Data are mean ± SEM. *denotes significant effect of ICP change while # denotes significant interactions effect (group vs. ICP change); p < 0.05.

Baseline levels of ICP, arterial pressure and renal blood flow in the control animals are shown in Table 1. ICV infusion of saline produced a ramped increase in ICP of 20 ± 1 mmHg over the 30-min infusion period (Figs. 1 and 2). The increase in ICP was accompanied by an increase in arterial pressure; the magnitude of which was almost matched to the increase in ICP (16 ± 2 mmHg). The increase in arterial pressure meant that the cerebral perfusion pressure was maintained. We also observed a significant decrease in renal vascular conductance as ICP increased which led to no change in renal blood flow (Fig. 2).

**Table 1:** Baseline levels of cardiovascular variables in normotensive and hypertensive animals.

	Normotensive	Hypertensive
Arterial pressure (mmHg)	86 ± 5	112 ± 5 *
Heart rate (bpm)	75 ± 4	81 ± 6
Intracranial pressure (mmHg)	7 ± 1	6 ± 2
Cerebral perfusion pressure (mmHg)	79 ± 5	102 ± 6 *
Renal blood flow (mL/min)	524 ± 52	659 ± 65
Renal vascular conductance (mL/min/mmHg)	6.5 ± 0.6	6.1 ± 0.8

Clipping of the renal artery was associated with a significant increase in arterial pressure but there was no change in baseline heart rate or renal blood flow to the contralateral kidney (Table 1). Baseline ICP levels in the hypertensive animals was not different to the normotensive animals which meant that cerebral perfusion pressure was significantly augmented in the hypertensive animals (Table 1).

ICV infusion of saline in the hypertensive group also increased ICP over the 30-min infusion period to similar levels as the normotensive group (19 ± 2 mmHg). However, the increase in arterial pressure was significantly attenuated in the hypertensive group (5 ± 2 mmHg) compared to the normotensive group. The attenuated increase in arterial pressure meant that cerebral perfusion pressure decreased in the hypertensive group during the ICV saline infusion. There was a similar decrease in renal vascular conductance with the increasing ICP which meant that renal blood flow was not altered during the ICV infusion.

### Intracranial baroreflex gain in the two groups

**Figure 3:**
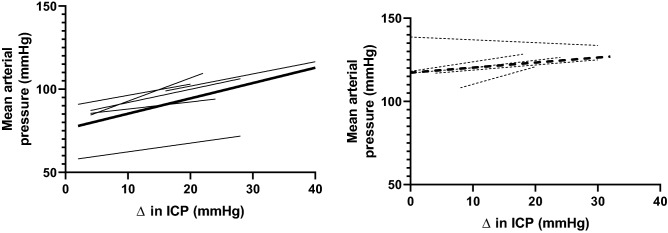
Average and individual regression lines showing the relation between changes in arterial pressure as a function of changes in intracranial pressure for normal (left panel) and hypertensive (right panel) groups. The thin lines are individual animals and the thick lines are the mean regression lines for each group.

The change in arterial pressure was plotted as a function of the increase in ICP in each animal. We refer to this reflex as the intracranial reflex and the slope of the individual lines was significantly altered in the hypertensive group (0.36 ± 0.1 in the hypertensive group vs. 0.85 ± 0.2 in the normotensive group; p < 0.05) indicating the intracranial reflex is attenuated in hypertension (Fig. 3).

**Figure 4:**
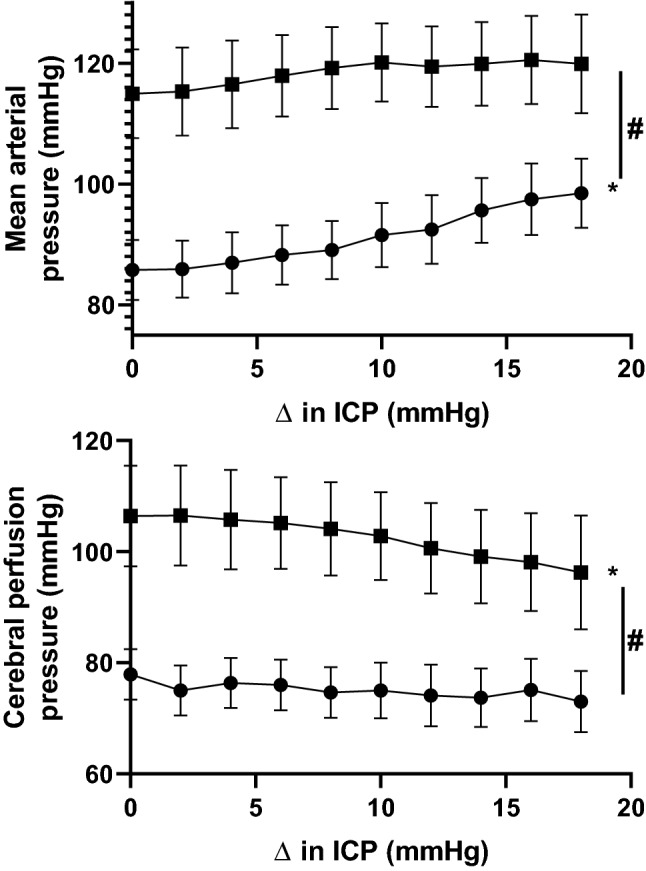
Mean response of arterial pressure and calculated cerebral perfusion pressure (CPP) to the increase in intracerebroventricular infusion of saline in the normotensive (filled circles; n = 6) and hypertensive (filled squares; n = 6) groups. Data are means ± SE and are plotted against change in intracranial pressure (ICP) from baseline. *Significant effect of time; # significant interaction between group and ICP (two-way ANOVA).

To determine if the lack of an increase in arterial pressure was related to the absolute cerebral perfusion pressure, the absolute levels of cerebral perfusion pressure and arterial pressure to the change in ICP were plotted (Fig. 4). Baseline levels of cerebral perfusion pressure were higher in the hypertensive group compared to the normotensive group (Table 1). While cerebral perfusion pressure decreased in the hypertensive group, the absolute level at the end of the ICP challenge was still higher than the baseline cerebral perfusion pressure in the normotensive group (Fig. 4).

### Increases in ICP during ganglionic blockade

**Figure 5:**
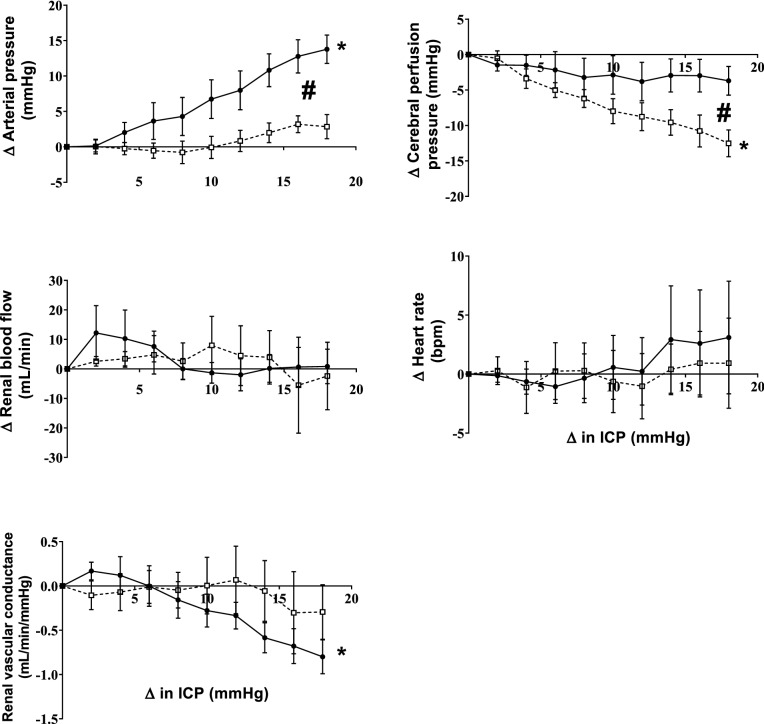
Response of arterial pressure, cerebral perfusion pressure, heart rate, renal blood flow and renal conductance to increased ICP in control sheep before (closed circles, n = 6) and after ganglionic blockade with hexamethonium (open circles, n = 6). Data are mean ± SE and are plotted as change from baseline. *denotes significant effect of ICP change while # denotes significant interactions effect (group vs. ICP change); p < 0.05.

Infusion of the ganglionic blocker hexamethonium chloride for two hours led to a significantly greater decrease in arterial pressure in the hypertensive group compared to the normotensive group (-15 ± 4 vs. -6 ± 1 mmHg). There was no change in ICP for either group. Ganglionic blockade abolished the increase in arterial pressure in the normotensive group to increased ICP and was therefore associated with a significant decrease in cerebral perfusion pressure (Fig. 5). Similar to the normotensive group, ganglionic blockade also abolished the increase in arterial pressure and this meant cerebral perfusion pressure decreased in response to the ICV infusion of saline in the hypertensive group.

### Comparison between the baroreflex and ICP infusion changes

**Figure 6:**
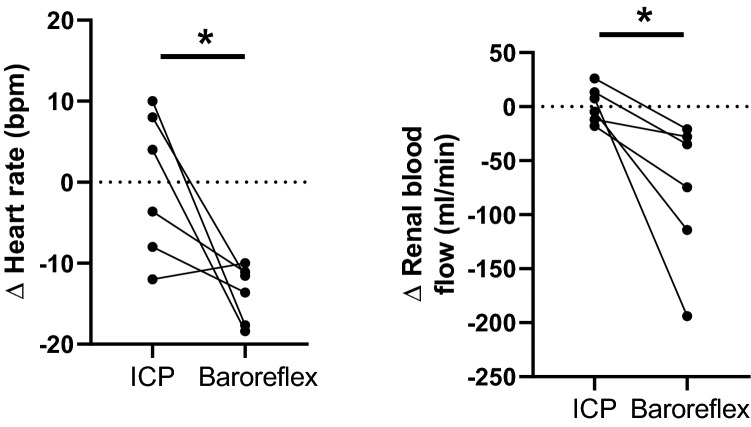
The change in heart rate and renal blood flow during the intracranial pressure challenge is compared to the changes during equivalent increases in arterial pressure during the baroreflex challenge in normotensive sheep. * significant difference between the two challenges.

The change in heart rate and renal blood flow during phenylephrine-induced changes in arterial pressure was plotted against the changes that occur during the ICV infusion in the normotensive group (Fig. 6). In the normotensive group, baroreflex mediated increases in arterial pressure led to significant decreases in heart rate and renal blood flow. For an equivalent increase in arterial pressure during the ICV infusion, there was no significant change in HR or renal blood flow. For the hypertensive group, the change in arterial pressure with ICV infusion was lower and hence there were no significant changes in heart rate or renal blood flow during the ICV infusion.

## Discussion

Our data in conscious sheep re-iterate our previous findings that modest physiological increases in ICP lead to an increase in arterial pressure that is mediated by the autonomic nervous system. Importantly, we now show that similar increases in ICP in hypertensive animals result in a severely attenuated response in arterial pressure. Consequently, increases in ICP lead to decreases in cerebral perfusion pressure in hypertensive animals. We have thus demonstrated that the reflex physiological mechanism that links levels of ICP with arterial pressure is attenuated in hypertension.

### Mechanism of the increase in arterial pressure in normal animals

We have previously shown that the increase in physiological levels of ICP results in an increase in directly recorded renal sympathetic nerve activity (SNA)^3^. The ICP-induced rise in BP was completely abolished during ganglionic blockade (Fig. 5) indicating this intracranial reflex is sympathetically mediated. Our data extends this to show that the increase in renal SNA causes a reduction in renal vascular conductance (Fig. 2). Other research groups have shown that muscle SNA is also increased suggesting vasoconstriction in the skeletal muscle bed as well^5^. Interestingly, there was no change in heart rate during the infusion. It is well known that sympathetic nerve activity is differentially controlled to different organs^17,18^ and while there may be increases in renal and muscle SNA, our data suggest that SNA to the heart is presumably not changed given no change in HR for the entire infusion period. The lack of a change in HR is also indicative that these changes in ICP are not pathological and do not activate the Cushing’s reflex. While an ideal scenario would be to determine the arterial pressure response to two different levels of ICP, it would be unethical to raise ICP levels to pathological levels in conscious sheep.

If the reflex increase in arterial pressure is related to the pressure itself, then the ICP needs to be sensed in the brain. Possible sites include neurons within the rostral ventral lateral medulla which are baro-sensitive^19^ or astrocytes around the parenchymal arterioles and capillaries which are mechanosensory^20^. Marina et al. elegantly showed that ICP can be directly sensed by astrocytes in the brainstem^4^. We also cannot rule out that the ICP may be sensed by the neurons lining the ventricle^21^. While these data suggest that the reflex is mediated by an increase in pressure within the cranium, it is also possible that the reflex may be related to reductions in cerebral perfusion (not pressure) although our indirect estimate of cerebral perfusion pressure suggests this is not the case.

In the normotensive animals, arterial pressure increases would trigger a baroreflex-mediated *inhibition* of the sympathetic nervous system. This suggests that during activation of the intracranial reflex, there must be attenuation of the baroreflex mechanism. A previous study has shown that the baroreflex is attenuated during large increases in ICP^22^. To explore this during physiological increases in arterial pressure in the normotensive animals, we directly compared the changes in heart rate and renal blood flow during the ICP challenge and the baroreflex challenge focusing on the same change in arterial pressure in each animal. Our data indicates that during the ICP challenge there is significant attenuation of the bradycardia that is seen during the baroreflex challenge (Fig. 6). Our data suggest that the baroreflex is presumably overridden by the excitatory mechanisms driving sympathetic nerve activity.

### Attenuation of the pressor response in hypertensive animals

Previous studies have hypothesized that this reflex increase in arterial pressure in response to elevated ICP may be altered in pathological conditions^2^. To the best of our knowledge, this is the first study to show that this is indeed the case. Interestingly the arterial pressure response to the initial increase in ICP (up to 8 mmHg) is very similar to the normotensive animals but then the increase in arterial pressure tapers off (Fig. 2). When the change in arterial pressure was plotted as a function of the change in ICP, the gain of this reflex was significantly attenuated in the hypertensive animals (Fig. 3) indicating changes in ICP do not lead to similar changes in arterial pressure. In a previous study we showed that in a group of ‘normotensive’ animals when the baseline arterial pressure was high, there was an attenuated response to the ICP challenge^3^. The attenuated rise in arterial pressure in that ‘non-responder’ group in the previous study is similar to that seen in the hypertensive group suggesting that both acute and chronic hypertension results in an attenuated intracranial baroreflex.

The model of hypertension used in this study is the 2 kidney 1 clip, which in sheep and dogs shows a very rapid rise in arterial pressure^23,24^. This is in contrast to a slower elevation of arterial pressure observed in rodents using this model. This means that in this study our model of hypertension had elevated arterial pressure for around 2–3 weeks before the ICP challenge was conducted. In this context, calculated cerebral perfusion pressure was higher in the hypertensive animals since there was no change in baseline levels of ICP. In contrast, hypertensive patients appear to have lower cerebral perfusion than their age-matched normotensive controls^9–11^. Whether the intracranial baroreflex is attenuated after months of hypertension remains to be determined. If the intracranial baroreflex is attenuated in chronic hypertensive patients, any increases in ICP would lead to decreases in cerebral perfusion pressure.

The reasons for the attenuation of the intracranial baroreflex in hypertension remain unclear. It cannot be related to an alteration in baroreflex control of sympathetic nerve activity to the heart or kidney since we have previously shown that this is not altered at this stage of hypertension in this model^25^. We were able to increase ICP to similar levels in the hypertensive animals so presumably the lack of a change is not related to an inability to increase the levels of ICP. We speculate that the attenuation may be due to inefficient transduction of the pressure to the neurons or astrocytes although this hypothesis remains to be tested.

### Limitations

It is important to consider the role of cerebral autoregulation in these animals and one of the limitations in this study is the absence of a measure of cerebral flow in these animals. The effects of the elevation in ICP on cerebral blood flow in the sham group would partially be offset by cerebral autoregulation. In this context, previous studies have shown attenuated cerebral autoregulation in hypertensive individuals^26,27^. In the hypertensive group, the presumed lack of cerebral vasodilation would lead to greater decreases in cerebral flow but this remains a speculation. One would expect a greater drive to increase sympathetic nerve activity and arterial pressure however, we observe an attenuated increase in arterial pressure. This could be due to two potential reasons. It is tempting to speculate that perhaps the postganglionic sympathetic nerve units are firing near their maximum level in hypertensive animals, and therefore do not allow for further recruitment. Our data, that arterial pressure decreases to a greater extent in response to hexamethonium in the hypertensive group, is supportive of this possibility although this remains to be directly tested. Alternatively, the vasculature response to heightened sympathetic drive may be attenuated as has been reported in animal models of hypertension^28,29^. While we have shown an attenuated arterial pressure response, direct recordings of renal sympathetic nerve activity would have further clarified why the response is attenuated.

In summary, we have shown that physiological increases in ICP lead to increases in arterial pressure in normotensive animals but this is attenuated in renovascular hypertension. An over-active sympathetic nervous system is involved in the initiation and maintenance of a number of cardiovascular diseases including hypertension, heart failure and chronic kidney disease^30–35^. It is known that the reflex regulation of arterial pressure by the arterial baroreflex and the peripheral chemoreflex is altered in hypertension. Our data indicate that in addition to these other regulators, the intracranial baroreflex regulation of arterial pressure is also attenuated in hypertension.

## Methods

All animal experiments were approved by the University of Auckland’s Animal Ethics Committee. All experiments were performed in accordance with the guidelines and regulations of the Ethics committee and these comply with the ARRIVE guidelines. Dry ewes (50-70 kg; 3–4 years old), acclimated to a standard pellet diet and the laboratory for at least 1 week prior to undergoing surgery. All sheep (n = 12 in total) were housed in individual metabolic home crates with multiple sheep in the same room, and constant visual contact with one another, at a constant temperature (18 °C) and humidity during an automated 12–12 h dark–light cycle (lights on from 0600 to 1800). Food and water were supplied ad libitum, except for the short duration during intracerebroventricular (ICV) infusion experiments when food and water were removed to minimize changes in ICP due to head movements. All experimental protocols are similar to that described by us previously^2^. The data that support the findings of this study are available from the corresponding author upon reasonable request.

### Surgical procedures

Experiments were conducted in a normotensive (n = 6) and a hypertensive (n = 6) group of animals. There was no difference in age between the two groups of animals. Both groups underwent two aseptic surgical procedures prior to experimentation. General anesthesia was induced by i.v. thiopental sodium (15 mg/kg) and maintained by (2–3%) isoflurane following intubation. Intraoperatively, sheep were treated with intramuscular antibiotics (20 mg/kg oxytetracycline).

In the first surgery, for the hypertensive group the renal artery was exposed and isolated through retroperitoneal incision, and a metal clip was tightened around the artery to reduce blood flow to around 40% of resting values. In the sham normotensive group, the renal artery was exposed but no clip was placed. The second surgery was performed two weeks after the clip/sham surgery. To allow measurement of ICP and arterial pressure, two 3.5 Fr solid state pressure catheters (SPR-524, Millar, Houston, TX) were implanted into the carotid artery for measurement of arterial pressure and subdurally for measurement of ICP. The cranium was sealed around the ICP catheter using Gelfoam and dental impression material. One stainless steel guide tube was inserted so the tip was 5 mm above the lateral cerebral ventricles. This served as the ICV cannula. The ICV cannula and ICP catheter were secured in place using stainless steel screws and dental cement. A jugular catheter was also inserted for intravenous infusion and drug administration. The renal artery was isolated using a retroperitoneal incision and a Transonic flow probe (Transonic, 6PS) was placed around the renal artery to measure renal flow as described previously^36,37^. Note that renal flow probe in the hypertensive animals was instrumented in the renal artery contralateral to the clipped kidney. Post-operative analgesia (ketofen, 2 mg/kg, Boehringer Ingleheim Ltd, Auckland, New Zealand) was given as needed. To minimize any effect of surgical stress, the sheep were allowed to recover for at least 4 days before ICV infusion experiments were started. By this time point, the animals had resumed normal eating and drinking and baseline variables were similar to previous reports of unstressed animals.

### Data acquisition

On the day of each experiment, the ICP and arterial pressure pressure catheters were connected to a dual channel pressure control unit (PCU-2000, Millar Inc, Houston TX) and the renal flow probe (RBF) was connected to the Transonic flow meter. The ICP, arterial pressure and RBF signals were recorded using PowerLab and LabChart (ADInstruments, v8.15, Sydney, Australia) at a sampling frequency of 1 kHz. A webcam was used to video the sheep and monitor their movements. This video was captured by LabChart synchronised with the ICP, arterial pressure and RBF recordings, and was used to discard any transient changes in ICP due to head movement as done previously by our group^3^.

### Intracerebroventricular (ICV) infusion

On the day of the experiment, a sterile inner cannula was inserted such that the tip of this cannula was in the lateral ventricles. The flow of cerebrospinal fluid in and back out of the ventricle confirmed placement of the ICV cannula. To increase ICP, sterile saline was infused via the ICV cannula using a syringe pump (Harvard apparatus, USA). The infusion started at a rate 5 ml/hr and was doubled every 10 min until the mean ICP was increased by 15–20 mmHg above the baseline (~ 30–45 min). The ICV infusion was stopped if behavioural changes (head position, panting etc.) were noted.

To determine the involvement of the sympathetic nervous system, the ICV infusion was repeated on a different day after a 2-h infusion of ganglionic blocker hexamethonium chloride (125 mg/hr) which has been shown to block all sympathetic tone^38^. Hexamethonium infusion was then continued throughout the ICV infusion. There was at least 24 h between these different infusions and the order was randomized for all the animals.

### Baroreflex curves

To compare the changes in heart rate and renal blood flow when arterial pressure is increased, the baroreflex was investigated during infusion of phenylephrine (Sigma-Aldrich, St Louis, MI) at escalating doses (0.02 up to 0.9 mg/minute).

### Data analysis

Cerebral perfusion pressure was calculated as the difference between arterial pressure and ICP. Heart rate was derived from the arterial pressure waveform using LabChart’s cyclic measurement feature.

The analysis of this data has been described previously^2^. Briefly, ten-second averages of ICP, arterial pressure, HR and CPP were calculated throughout the ICV infusion period. In Excel, the 10 s averages were sorted into 2 mmHg ICP bins from baseline to the maximum change in ICP and the mean of the binned data calculated for each variable. The change in each variable from baseline was calculated and expressed against the change in ICP from baseline. To determine if the changes in heart rate or renal blood flow during ICV infusion of saline were related to the change in arterial pressure, the corresponding change in heart rate or renal blood flow during baroreflex challenge was calculated for each animal. Renal vascular conductance was calculated as the renal blood flow values divided by arterial pressure.

### Statistical analysis

All results are reported as mean ± SEM. Unpaired t-tests were used to compare differences between baseline values for the different groups in Table 1. Paired t tests were used to compare heart rate and blood flow changes in Fig. 6. For time series data, univariate ANOVA was utilized. The response of each variable to increased ICP was determined using an ANOVA with levels of ICP, and individual animals included as factors. The effects of ICP levels as well as the interaction term between levels of ICP and group were focused on. Linear regression was used to determine if the slope of the arterial pressure response to increased ICP was different between groups in Fig. 3. In all cases *p* < 0.05 was considered significant.
